# The crucial roles of inflammatory mediators in inflammation: A review

**DOI:** 10.14202/vetworld.2018.627-635

**Published:** 2018-05-15

**Authors:** L. A. Abdulkhaleq, M. A. Assi, Rasedee Abdullah, M. Zamri-Saad, Y. H. Taufiq-Yap, M. N. M. Hezmee

**Affiliations:** 1 Department of Pathology and Poultry Diseases, Faculty of Veterinary Medicine, Baghdad University, Baghdad, Iraq; 2 Department of Veterinary Pathology and Microbiology, Faculty of Veterinary Medicine, Universiti Putra Malaysia, Malaysia; 3 Department of Community Health, College of Health and Medical Techniques, Al-Furat Al-Awsat Technical University, Iraq; 4 Department of Veterinary Preclinical Sciences, Faculty of Veterinary Medicine, Universiti Putra Malaysia, Malaysia; 5 Department of Veterinary Laboratory Diagnostics, Faculty of Veterinary Medicine, Universiti Putra Malaysia, Malaysia; 6 Department of Chemistry, Faculty of Sains, Universiti Putra Malaysia, Malaysia

**Keywords:** chemokines, cytokines, inflammatory mediators, inflammatory response

## Abstract

The inflammatory response is a crucial aspect of the tissues’ responses to deleterious inflammogens. This complex response involves leukocytes cells such as macrophages, neutrophils, and lymphocytes, also known as inflammatory cells. In response to the inflammatory process, these cells release specialized substances which include vasoactive amines and peptides, eicosanoids, proinflammatory cytokines, and acute-phase proteins, which mediate the inflammatory process by preventing further tissue damage and ultimately resulting in healing and restoration of tissue function. This review discusses the role of the inflammatory cells as well as their by-products in the mediation of inflammatory process. A brief insight into the role of natural anti-inflammatory agents is also discussed. The significance of this study is to explore further and understand the potential mechanism of inflammatory processes to take full advantage of vast and advanced anti-inflammatory therapies. This review aimed to reemphasize the importance on the knowledge of inflammatory processes with the addition of newest and current issues pertaining to this phenomenon.

## Introduction

The inflammation term is taken from the Latin word “inflammare” (to burn) (*de oliveira*). Inflammation is one of the most central processes required in defense of animal cells against certain injuries or microbial infections [1,2]. Nevertheless, inflammation regularly progresses to acute [3] or chronically [1]. Chronic inflammation is caused due to a variety of diseases including neurodegenerative disorders, cancer, and cardiovascular diseases [4].

Mechanism of inflammation represents a chain of organized, dynamic responses including both cellular and vascular events with specific humoral secretions. These pathways involve changing physical location of white blood cells (monocytes, basophils, eosinophils, and neutrophils), plasma, and fluids at inflamed site [5]. A group of secreted mediators and other signaling molecules (e.g., histamine, prostaglandins, leukotrienes, oxygen- and nitrogen-derived free radicals, and serotonin) are released by immune defense cells principally in the mechanism which can contribute in the event of inflammation [6].

Whatever, the inflammatory response is triggered through two phases: (a) acute and (b) chronic, and each is apparently mediated by a different mechanism [3]. These immune responses which involved in acute inflammation can be divided into vascular and cellular [7].

The responses which occur in microvasculature normally appear in few minutes following tissue injury or microbial infection in the presence of other inflammatory stimuli named vascular events [7]. The occurrence of these processes is rapid and eventually will lead to vasodilation and subsequently makes the vessels become more permeable. This processes will result in entry of inflammatory mediators and produces interstitial edema [8].

Inflitration of white blood cells from circulatory system is essential during inflammatory responses [9,10]. A group of chemotactic agents such as microbial endotoxins holding amino terminal N-formyl methionyl groups, C5a complement fragment, and interleukins along with the secretions of basophils such as platelets activating factor, histamine, and leukotriene B can stimulate intense leukocytes infiltration within few minutes [11,12]. Among the leukocytes, neutrophils are the first inflammatory cells that are recruited at the acute inflammation site [13]. Infiltration of immune cells triggered via a complicated mechanism in which white blood cells work together with endothelium in postcapillary venules [14].

Cellular events encompass the successive capture, trundling, and firming an adhesion to the microvascular endothelium [15]. These events in the mobilization pathway are arranged by cell adhesion molecules (CAMs). These CAMs include intracellular adhesion molecules (ICAM)-1, ICAM-2, integrins, and selectin. The selectin group of CAM contains three families; P-selectin and E-selectin produced by endothelial cells and L-selectin produced by white blood cells [16].

The adhesion of high affinity presented on white blood cells in the endothelium is mediated by the interaction between integrins (CDII/CDI8), and adhesion molecules (CAM-l and CAM-2) expressed on white blood cells and endothelium cells, respectively [17]. Following a period of stationary adhesion, the white blood cells may leave the postcapillary venules extending pseudopodia between endothelial cells and reach into the subendothelial space. This complex event is often referred as white blood cell extravasations and transendothelial migration [18].

The inflammation of chronic events are distinguished by mononuclear cell infiltration (e.g., monocyte and lymphocytes), fibroblasts proliferation, collagen fibers, and connective tissue formation, which ultimately result in 2-mm granuloma [19]. With chronic inflammation, the tissue degeneration is normally mediated by nitrogen species, proteases, and other reactive oxygen species released from infiltrated inflammatory cells [20]. Certainly, genomic alterations in p53 were approved as causes for many chronic inflammatory diseases (e.g., inflammatory bowel diseases and rheumatoid arthritis) in addition to cancers [21-23].

The novelty of this review is that it provides the summary of the latest accumulation of knowledge about the involvement of mediators in inflammation while untangling some misconception and argument regarding the inflammatory processes.

This review aimed to reemphasize the importance of the knowledge of inflammatory processes with the addition of newest and current issues about this phenomenon.

## Mediators

A variety of chemical mediators from circulation system, inflammatory cells, and injured tissue actively contribute to and adjust the inflammatory response [24]. The released chemical mediators include (1) vasoactive amines such as histamine and serotonin, (2) peptide (e.g., bradykinin), and (3) eicosanoids (e.g., thromboxanes, leukotrienes, and prostaglandins).

## Vasoactive Amines and Peptide

Histamine is released in a quantity of few pictograms from basophils to maintain acute-phase response during inflammation events [25].

Serotonin is produced via decarboxylation of tryptophan, and it is stored in the granule [26]. In murine, the serotonin is available in basophilic granules, while in humans, it is present in platelets. Four serotonin receptors, namely 5-HTl, 5-HT2, 5-HT3, and 5-HT4, were documented to mediate its biological functions [27].

Bradykinin is a nanopeptide created from plasma Kinin–Kallikrein system [28]. Two or more distinct receptors are present for bradykinins which have been titled B1 and B2 [29]. Similar to histamine and serotonin, it can increase the synthesis of prostaglandins and produces pain locally [30].

## Eicosanoids

Arachidonic acid, which represents the main component of membrane phospholipids in all the cells, is one of the most important substrates in the synthesis of biologically active mediators of the inflammation called eicosanoids [31]. The latter includes the products of 5-lipoxygenase (leukotriene and 5-hydroxyeicosatetraenoic acid), cyclooxygenases (prostaglandins and thromboxanes), and 12-1ipoxygenase (12-hydroxyeicosatetraenoic acid) [32,33].

The 5-lipoxygenase enzyme was discovered in 1976 from glycogen-elicited rabbit polymorphonuclear leukocytes [34]. The production of 5-LOX protein is mainly created in the immune cells of myeloid origin: (1) mononuclear cells such as rhogocytes, necrophages, and lymphocytes [35] and (2) polymorphonuclear leukocytes such as neutrophils and eosinophils [36]. These cells display a vital role in immune responses inflammatory reactions. However, erythrocytes, platelets, endothelial cells, and T-cell are 5-LOX negative [37].

Cyclooxygenase is an enzyme involved in the synthesis of proteinoids including potent proinflammatory prostaglandins and metabolism of arachidonic acid, which exists in at least two isoforms: cyclooxygenase-1 and -2 [38]. Cyclooxygenase-1 is produced constitutively in most of the mammal cell types and platelets. It is also secreted in vascular endothelium, stomach, forebrain, uterine epithelium, and kidney.

On the other hand, cyclooxygenase-1 (not only cyclooxygenase-2) has a pathological role in the animal body, and it can also be stimulated at the site of inflammation [39].

These findings have been further supported by different models of carrageenan-induced inflammation. First, mice that are lacking the gene for cyclooxygenase-1 showed a diminished inflammatory reaction when a compared to wild-type. Secondly, mice that are lacking the gene for cyclooxygenase-2 showed the inflammatory response of similar magnitude to those observed in wild-type. Therefore, these results have indicated that cyclooxygenase-1 participates in the onset of inflammation along with cyclooxygenase-2 [40].

Prostanoids, formed by cyclooxygenase-l, are important in many physiological functions including regulation of platelet aggregation as thromboxane-2 induces platelet aggregation while PGh exhibits antiaggregatory properties [41]. In the alimentary canal, prostaglandin-h and prostaglandin E2 inhibit secretion of gastric acid, employ an uninterrupted vasodilator effect on the blood arteries and veins of the gastric mucosa, and induce the viscous mucus creation which represents a protective barrier [42]. In the kidney, vasodilator prostaglandins (prostaglandin-h, prostaglandin E2, and prostaglandin D2) account for a significant portion in dilating of renal vascular beds, improving organ perfusion, regulating of renal blood flow, and shrinking of vascular resistance [43,44].

Cyclooxygenase-1 is produced by neuronal cells in all parts of brain. However, forebrain, where the prostaglandins are needed for complex integrative functions thereby this enzyme, is produced abundantly [45]. Cyclooxygenase-1 is also produced in the uterine epithelium in the first stages of pregnancy and could be significant to enhance the ovum and for the placenta formation and angiogenesis requirements [46].

Meanwhile, prostaglandins (prostaglandin E2 and prostaglandin b) are substantially encompassed in conserving the inflammatory process by increasing the vascular permeability and strengthening the outcome of other inflammatory mediators such as kinin, serotonin, and histamine and thus contributing to the redness, increased blood flow, and plasma exudation in the area of acute inflammation which leads to edema [47]. These prostaglandins produce hyperalgesia by affecting the afferent C fibers. Furthermore, prostaglandin E2 acts on neurons in the thermoregulatory network of the hypothalamus, causing an increase in body temperature [48].

**Figure-1:**
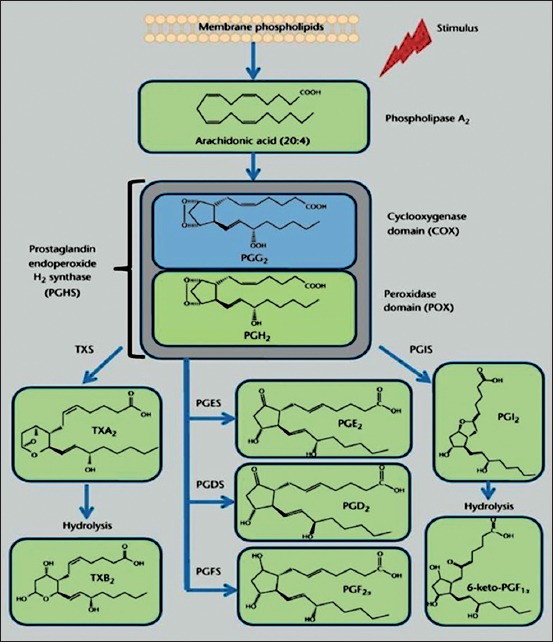
The cyclooxygenase pathway of the arachidonate cascade. In response to chemical and mechanical stimuli, arachidonic acid, a 20-carbon fatty acid with four double bonds (20:4), is released from membrane phospholipids by phospholipase A_2_. Prostaglandin endoperoxide H_2_ synthase (PGHS) catalyzes the bis-oxygenation of free AA into the unstable endoperoxide PGG_2_ and the reduction of PGG_2_ into PGH_2_, by the coordinated activity of the cyclooxygenase (COX) and the peroxidase domain (POX). PGH_2_ is further metabolized by cell-specific terminal isomerases and reductases to yield prostanoids. TXS, thromboxane (Tx) A_2_ synthase; PGDS, prostaglandin (PG) D_2_ synthase; PGES, prostaglandin (PG) E_2_ synthase; PGFS, prostaglandin (PG) F_2a_ synthase; PGIS, prostaglandin (PG) I_2_ synthase. TXA_2_ and PGI_2_ are unstable metabolites and hydrolyzed within minutes from their synthesis into the inactive metabolites, TxB_2_ and 6-keto-PGF_1α_ (Adapted from[51]).

Elevated levels of multiple prostaglandins including prostaglandin E2 and prostaglandin b have been reported in synovial fluids from patients with rheumatoid arthritis and osteoarthritis [49]. Prostaglandins also play an important role in the pathogenesis of several types of cancers such as breast, liver, and lung with overexpression of cyclooxygenase-2 and overproduction of prostaglandin (Figure-1) [50].

## Proinflammatory Cytokines

In addition to many stromal cells, fibroblasts, and endothelial cells, every cytokine can be released from many cells types [52].

The metabolic, hormonal, and physiological alterations increase the form power of the most important medical features [53]. These symptoms include weight loss, fever, and anorexia [54].

Cytokines have important effects in the activity of many cells. However, they are of particular importance because of their significance in regulating the immune system [55]. The function of cytokines in the manner of development in inflammatory disease as a result to bacterial infection or exposure to lipopolysaccharide (LPS) was investigated deeply in animals [56], pigs [57], cattle [58], and mice [59].

In addition, the production of cytokines induces the release of acute-phase response. Interleukin (IL)-1β, IL-8, tumor necrosis factor alpha (TNF-α), IL-6, and IL-12 are the most remarkable secretions included in these reactions [53]. The generation of animal toxicity is mainly attributable to secretion of IL1β, IL-6, and TNF-α as a result of exposure to LPSs of pathogens [60]. Not only LPSs have elevated interleukins secretions, but also they caused neuroinflammation in the infected animals [54].

Based on the infection route, particularly, inflammatory response can be successful to get rid of the causes of the disease [61]. In such case, this response is acute (short-term) and limited to the area where tissue damage occurs [59]. That will lead to an increase in macrophage-derived cytokine density in the plasma. These cytokines affect other organs, especially the brain and liver, resulting in a systemic immune response called the acute-phase response [61].

## Acute-phase Proteins

The interleukins have a strong effect on liver cells and stimulate them to create a class of proteins named acute-phase proteins [62]. It was found that acute phase-proteins in serum in normal and healthy person are at the basal concentrations. However, their levels are increased during liver stimulation [63]. Based on their elevation degree, acute-phase proteins are divided into two categories. The rise in the concentration of some acute phase proteins ranged from 1-fold to 1.5-fold while the others raised up to 1000-fold as seen down [64].

### Acute-phase proteins which raise from 1.5- to 5-fold

#### Fibrinogen

It has a vital role in fibrinopeptides generation and clotting [64].

#### Haptoglobin

It can combine to iron-containing hemoglobin and decrease the levels of iron which bacteria need for its metabolism, in that way it decreases its growth [64].

#### Complement component C3

It is normally cleaved to produce C3a, which excites the basophilic cells, and C3b, which aid phagocytes to identify pathogens [64].

#### Mannose-binding protein (MBP)

It binds to mannose-containing sugars, lying on the surface of a microorganism, and it makes it easier for phagocytes to identify pathogens [64].

### Acute-phase proteins which rise from 100- to 1000-fold

#### Serum am yloid A

This protein reduces platelet activation and fever, and by itself, it gives a vital negative feedback control loop in the common physiological systems [65].

#### C-reactive protein (CRP)

This protein can combine to phosphorylcholine, which is available on the surface of a microorganism, and it is shown in the injured cells. CRP assists phagocytes to identify pathogens or damaged cells [66].

In parallel to that, the blood flow and the permeability in vascular system are raised up due to inflammatory mediators [67]. These proteins offer supplementary factors which aid in the elimination of bacteria [68]. MBP [69] and CRP [61] are three central acute-phase proteins which work as opsonins to aid phagocytes to identify pathogens.

That elevation in haptoglobin and serum amyloid A was lowest with those group inoculated by whole bacteria. A significant increase has been detected in animals inoculated outer membrane proteins [70].

The animals inoculated with LPS showed the highest concentrations in both haptoglobin and serum amyloid, and that is attributable to its high toxicity and long immunogenicity which induce wide injuries in the tissues [71]. The mice inoculated with Gram-negative bacteria, and its LPS showed great elevation in haptoglobin and other acute-phase proteins. The suspicious role of sharp induction for acute-phase proteins in inflammation, neuronal necrosis, and cerebral vascular congestion has been deeply in murine [72].

## Monocytes (Macrophages)

This distribution enables monocytes well suited to exert a strong defense against foreign and their endotoxin earlier than white blood cells migration [73].

Monocytes are recognized as the most principle immune effector cells [74]. Monocytes are available in fundamentally all tissues [75]. They can differentiate, in the process of growth or development, from the peripheral mononuclear cells in blood circulating system and move to any cells in the “steady state” and/or in reaction to inflammatory induction [76]. The peripheral mononuclear cells are originated in the bone marrow from the common myeloid progenitor cells (precursor of many different cell types) to neutrophils, eosinophils, and basophils [77].

**Figure-2:**
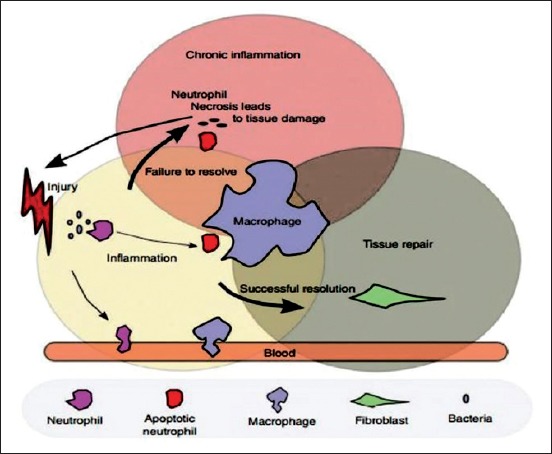
Macrophage role in inflammation and tissue repair. Upon stimulus, monocytes and resident macrophages activate. They remove tissue debris and produce inflammatory signals that promote the inflammatory response. Macrophages produce a wide array of cytokines, chemokines, and growth factors that promote inflammation, its regulation, and the successful restoration of tissue. They also participate in the regulation of inflammation by removing apoptotic neutrophils, an important process in turning the inflammatory process to one of tissue replacement and remodeling, apoptotic neutrophils that are not removed can undergo necrosis, spilling their toxic content, and perpetuating the inflammatory response (Adapted from [78]).

The latter is released into blood circulation from its manufacturer (bone marrow) after some sophisticated steps (Figure-2) [78,79].

Monocytes possess a significant role in both adaptive and innate immunity through their interacting with many immunological and non-immunological cells to trigger feat inflammatory response and clearance of foreign elements [80]. Intrinsically, monocytes play a central role by interacting with immune cells including T-lymphocyte cells, neutrophils, fibroblasts, B-lymphocyte cells, dendritic cells, and natural killer cells [81] (Figure-2). In relation to monocyte activation and phagocytosis, a huge number of monocyte researches have demonstrated the stimulation of cytokines such as TNF-α, IL-1β, IL-6, IL-10, as well as the transforming growth factor [82]. Reactive nitrogen species, macrophage inflammatory protein-2 [83], nitric oxide, monocyte chemoattractant protein-1, and reactive oxygen species are chemokines generated commonly as a response to monocyte activation and phagocytosis [84].

When the microbial endotoxin (such as LPS) contacts, several signaling pathways are concurrently stimulated to determine the phagocyte response [85] as well as control the internalization process of foreign elements by monocyte (macrophage) [86]. The phagocytosis is a sophisticated immune response with special highlighting on four reasons of this complexity: (1) Numerous different receptors interact with foreign elements, and phagocytosis is typically mediated instantaneously by many receptors [87], (2) dissimilar microbe-recognition supportively (or occasionally destructively) to trigger definitive responses to invaders [88], (3) the microbe recognition is directly coupled by phagocytic receptors or indirectly coupled by coreceptors to inflammatory events, which in its turn, regulate the effectiveness of foreign elements internalization through either phagocyte or neighboring phagocytes [89], (4) a lot of microbial elements actively contribute to regulate the phagocytosis mechanisms to avoid destruction. Phagocytosis also is essential for healthy clearance of apoptotic bodies, a process of programmed cell death [89].

Loads of signaling mediator such as lipases, membrane traffic regulators, kinases, actin-binding proteins, and ion channels are stimulated in the course of phagocytosis for opsonized microbe (or complex particles such LPS) and can lead to successful internalization [90].

Conversely, some signaling proteins (and molecules) contribute to two immune mechanisms: (1) phagocytosis mechanism and (2) dozens of other signaling pathways. Rho GTPases, phospholipase C, and phosphoinositide 3-kinase are not just mediate the ingestion process of foreign elements [91].

The produced phospholipid is important in enrolling some signaling mediators (e.g., kinase AKT/PKB) to a certain area of cell membranes [92]. Inhibition of phosphoinositide 3-kinase blocks phagocytosis process against microbes, unopsonized zymosan, and complement- and immunoglobulin G-opsonized particles [93]. Phosphoinositide 3-kinase inhibition leads to block the extension of the cell membrane, and then, its fusion behind the bound foreign materials is attributed to a disability to insert new membrane at the site of foreign material internalizations [94].

Monocytes use both the pattern recognition receptors (e.g., Toll-like receptors) to trigger immune defenses [68]. Thus, the whole collection of immune responses and the inflammatory cytokine promoter and IL-6 promoter are activated [68]. Certainly, many bacteria have capability to survive in spite of cytokine production [95].

Although the process of destruction of microbial agents is triggered by the release of reactive oxygen species [96], in some instance, some phagocytic elements which cannot employ Toll-like receptors mechanisms will have the LPSs upregulation of the phagocyte oxidase [97].

The mechanism of phagocytosis needs the employment of actin filaments during internalization response [98]. The generation of reactive oxygen species can work as a second messenger, and it can also trigger different signaling pathways [99]. These pathways lead to the induction of nuclear factor kappa-B (NF-κB) causing the expression of pro-inflammatory interleukins (e.g., IL-6) and TNF-α[99].

## Anti-inflammatory Drugs

The main anti-inflammatory drugs are either steroidal [100] (e.g., betamethasone, prednisolone, and dexamethasone) or nonsteroidal [101] (e.g. aspirin, diclofenac, ibuprofen, indomethacin. naproxen, nimesulide, and celecoxib) used to treat both acute inflammatory condition and chronic inflammatory diseases such as osteoarthritis and rheumatoid arthritis [102].

However, their prolonged use is associated with various side effects; for example, steroidal drug causes adrenal atrophy [103], osteoporosis, suppression of response to infection or injury, euphoria. Cataracts, glaucoma, and non-steroidal drug [104] cause peptic ulcers and bronchospasm due to blockade of both the physiological and inflammatory prostaglandins and concurrent production of leukotrienes.

Thus taking into account the adverse effects [105] and high cost of synthetic conventionally available steroidal or non-steroidal drugs [106], the search for new anti-inflammatory agents from herbal sources is getting popular with the objective to obtain greater safety, better efficacy, and a more economical way to treat inflammation.

## Natural Products in Anti-inflammation

### Natural product

For 1000 years, the medications were totally of natural origin and extracted from inorganic materials, plant and animal products [107]. Primary remedies can commonly have combined these components with mysticism, witchcraft astrology, or religion; however, it is assured that those medications, which were in effect, were successively verified and recognized, leading to the early herbalist [108].

Herbal medicine continues to be an accepted form of treatment in the Orient, and plant drugs based on traditional practice represent a huge portion of the pharmaceutical products in modern western countries [109].

First, concerns have been raised that modern pharmaceutical practice too often involves costly drugs that produce unacceptable side effects [110]; second, the experience shows that natural substances can apparently address several modern health concerns with fewer side effects [111]; and third, experience shows that modern medicine and traditional herbal medicine can be combined [112].

Moreover, there are countless cause’s make publics take herbal medications, including the acceptance that natural is better, fear or distrust of physicians, disappointment with allopathic care, and cultural or religion influences [113].

Interest in the use of natural bio-resources to manage chronic diseases such as cancer has been increasing in recent years [114]. It is attributed to issues of side effects and prices of pharmacological therapies.

## Anti-inflammation

Approximately 75% of the population through of this world relies on traditional medications of herbal origin for health care purpose as reported by the World Health Organization [115]. The plants (or herbs) represent humanity as the eldest friends [116]. They are not the only source of food (or shelter) but have also aided the humankind to cure several diseases [117]. The herbal medicines are traditional (or natural) medicine applied by many people of different traditions and civilizations as approved by Mesopotamians, Egyptians, Greco-Arab, and Chinese [118].

Ayurveda and Chinese medicinal systems are the most acceptable traditional systems which have an extensive focus on working on pharmacology [116]. About 80% of population in developing countries China, India, and Pakistan relies on traditional medicines which make this region different from the West that has lost this tradition ill the process of modernization and rapid development in the last two centuries [119], and according to an estimate, only 25% of all prescriptions in the United States are from natural products [120].

Indeed, today, many pharmacological classes of drugs available in the market are derived from natural products prototype including atropine from *Atropa belladonna* (Solanaceae), reserpine from *Rauwolfia serpentina* (Apocynaceae), digoxin from *Digitalis purpurea* (Scrophulariaceae), theophylline from *Camellia sinensis* (Theaceae), morphine and codeine from *Papaver somnifera* (Papaveraceae), quinine from *Cinchona officinalis* (*Rubiaceae*), taxol from TaXI/X brevifolia NUll, and vincristine and vinblastine from *Vinca rosea* (Apocynaceae) [121].

Nowadays, the interest in herbal compounds at a global level is revived, and this focus calls attention of many researchers and governments because the sales of natural products in the world have exceeded 0.1 trillion US dollars yearly [122]. For example, Germany is the first country in European Community (followed by France) in the usage of the substance obtained from a plant and used as an additive, especially in gin or cosmetics [123].

By ancient Greek physicians, the history of substances which are acting to relieve pain has arisen with plants or herbs containing salicylate [124].

Herbal ointments such as *Aloe vera* gel along with cortisone (hydrocortisone-21-acetate) enhance the anti-inflammatory activity in the skin, suggesting that it has an important role as a pharmaceutically active carrier for steroids [125]. In addition to that, 25% *A. vera* in Eucerin cream and 5% decolorized irradiated *A. vera* extract (containing anthraquinone) potently reduce wounds in mice [126]. On the basis of biological activity, *A. vera* is widely used as oral and topical preparation by podiatric physicians to treat inflammation and wounds of the foot [127].

## Conclusion

This review has highlighted the important roles of inflammatory mediators in the inflammatory process. Although inflammation is very important in the elimination of pathogens and other causes of inflammation, a prolonged inflammatory process has been shown to results in chronic disease processes that may eventually result in organ failure or damage. Thus, limiting the inflammatory process by the use of anti-inflammatory agents is important in controlling this process and limiting its course. However, while a handful of synthetic anti-inflammatory agents exist, they all seem to have adverse effects with prolonged usage. Hence, there is still the need to discover newer and better anti-inflammatory agents from natural products.

## Authors’ Contributions

LAA and RA drafted and edited the manuscript according to the title. MAA, MZ, YHT and MNMH contributed the references for the content and edited some portions in this manuscript. All authors read and approved the final manuscript.
